# Extracellular Particles Isolated from Leftover Discarded Formalin-Fixed Tissues Exhibit Atypical Extracellular RNA Profiles

**DOI:** 10.3390/biom16070993

**Published:** 2026-07-07

**Authors:** Vyshnavi Tallapaneni, Bryson C. Okeoma, Ravi Sachidanandam, Humayun K. Islam, Chioma M. Okeoma

**Affiliations:** 1 Department of Pathology, Microbiology and Immunology Basic Sciences Building, New York Medical College, 15 Dana Road, Rms 327, 328, 328A, Valhalla, NY 10595, USA; vtallapaneni@nymc.edu (V.T.); bokeoma@nymc.edu (B.C.O.); rsachidanandam@nymc.edu (R.S.); 2 Network Director, Pathology and Laboratory Services, Westchester Medical Center, Valhalla, NY 10595, USA; 3 Lovelace Biomedical Research Institute, Albuquerque, NM 87108, USA

**Keywords:** particle purification liquid chromatography (PPLC), formalin fixed tissues (FFT), extracellular particles (EPs), RNASeq

## Abstract

This study reports atypical findings indicating that extracellular particles (EPs) isolated from leftover discarded formalin-fixed tissues (FFTs) contain atypical RNA profiles that may be highly fragmented. Using particle purification liquid chromatography (PPLC), we isolated EPs from five tissue types that had been immersed in 10% neutral buffered formalin, stored at room temperature, and collected at the point of discard. Tissues were de-identified and maintained at room temperature until EP separation and exRNA purification. Purified EPs were stored at −80 °C before physicochemical characterization and small RNA sequencing. PPLC separation profiles were broadly consistent with expected EP signatures, although subtle shifts in physicochemical properties were observed. exRNA was successfully recovered from all samples; however, RNA-Seq analysis showed that most reads were unclassified, while others mapped to microbial taxa rather than host-derived transcripts. These results suggest that EP-associated RNA in FFT may be substantially degraded and that microbial particles or nucleic acids were present in the tissues despite formalin fixation. Microbial viability was not assessed, as this was outside the scope of the study. Nonetheless, several explanations are plausible, including RNA fragmentation due to prolonged formalin exposure, microbial contamination during pre-discard storage, or inadequate or ineffective fixation that permitted microbial persistence or enrichment. Collectively, these findings indicate that leftover discarded tissues that were not flash-frozen or properly stored may be unsuitable—or at minimum require caution—when used as a source of EPs or EP-associated RNA. Investigators should consider RNA degradation/fragmentation, microbial confounders, and fixation-related artifacts when interpreting EP or exRNA data derived from FFT.

## 1. Introduction

Extracellular particles (EPs) are a heterogeneous and biologically active class of cell-derived entities that have emerged as central mediators of intercellular communication in both physiological and pathological contexts [1,2,3]. EPs encompass classical membrane-enclosed extracellular vesicles (EVs)—including exosomes, microvesicles, and apoptotic bodies—as well as non-vesicular extracellular particles (NVEPs), previously described as extracellular condensates (ECs) [4,5]. Collectively, these particles transport nucleic acids, proteins, lipids, and metabolites that reflect the molecular state, lineage, and environmental history of their cell of origin. As such, EPs provide a stable, information-rich snapshot of tissue biology and have become powerful tools for interrogating disease mechanisms, identifying biomarkers, and developing therapeutic delivery systems [6,7,8,9,10,11].

Over the past decade, EPs have been isolated and characterized from a wide range of biological fluids, including blood plasma, urine, saliva, cerebrospinal fluid, semen, and cell culture supernatants [4,6,12,13,14,15,16,17,18,19,20,21,22,23,24,25,26]. More recently, tissue-derived EPs, including brain- and gastrointestinal tract derived EPs [4,5,26,27] have gained attention because they capture spatially contextualized molecular information that is often lost when analyzing dissociated cells or bulk tissue homogenates. Tissue-derived EPs retain molecular cargo assembled in vivo and therefore provide insight into cellular communication networks operating within intact organs [5,27,28,29,30]. However, the majority of tissue-based EP studies have relied on fresh or frozen specimens [31], which poses practical limitations for large-scale, retrospective, and longitudinal investigations.

It has been established that frozen tissues and archival specimens have untapped potential and limitations. Fresh-frozen tissues are widely considered the gold standard for molecular analyses, including RNA-sequencing and proteomics [32]. Nonetheless, their availability is often restricted by logistical, ethical, and economic constraints. Cryogenic storage requires continuous ultra-low-temperature maintenance, specialized infrastructure, and significant long-term costs [33]. Moreover, frozen tissues are typically limited to prospectively collected samples, thereby excluding the vast repositories of clinically annotated specimens stored in pathology archives worldwide.

In contrast, formalin-fixed tissues (FFT), frequently embedded in paraffin as formalin-fixed paraffin-embedded (FFPE) blocks, represent one of the largest and most systematically curated biological resources available for biomedical research. Pathology departments routinely archive FFPE tissues for diagnostic, legal, and quality assurance purposes, resulting in collections that span decades and encompass diverse organs, disease states, treatment histories, and patient outcomes [34,35,36].

Formalin fixation is the most widely used tissue preservation method in clinical pathology due to its ability to maintain cellular and tissue architecture and maintain molecular integrity. Formaldehyde penetrates tissues rapidly and reacts with amino, imino, and amide groups in proteins and nucleic acids, generating reversible and irreversible crosslinks that stabilize cellular structures [36]. During fixation, formaldehyde forms methylene bridges between proteins, as well as protein–nucleic acid and nucleic acid–nucleic acid complexes, effectively “locking” biomolecules in place. While these chemical reactions preserve morphology, they compromise molecular integrity. RNA isolated from FFPE tissues is typically fragmented, chemically modified, and covalently crosslinked, limiting its compatibility with many downstream molecular assays [37]. Multiple studies have shown that total RNA extracted from FFPE samples is often degraded to fragments averaging between 100 and 300 nucleotides in length [38,39]. Additionally, formalin introduces mono-methylol (-CH_2_OH) adducts to RNA bases—particularly adenine and cytosine—which interfere with reverse transcription and amplification [38]. Despite these challenges, advances in antigen retrieval and nucleic acid de-modification techniques have demonstrated that a substantial fraction of formalin-induced RNA damage is reversible. Heat-mediated reversal of crosslinks in mildly alkaline buffers can remove formaldehyde adducts without extensive additional degradation, significantly improving RNA performance in downstream applications [40,41]. These observations have reinvigorated interest in exploiting FFPE tissues for molecular analyses beyond basic histopathology.

If EPs and their associated RNAs could be reliably isolated from fixed tissues, the EP-associated RNAs would serve as robust molecular readouts that would provide unprecedented opportunities for retrospective mechanistic studies and biomarker discovery. Among RNA species, small RNAs—including microRNAs (miRNAs) are well suited for analysis from fixed tissues, because their short length (typically 18–30 nucleotides) renders them less susceptible to fragmentation-based loss, and numerous studies have demonstrated that miRNA expression profiles from FFPE tissues closely mirror those obtained from matched frozen specimens [42,43,44,45].

It is also expected that EP-associated RNA isolated from FFPE tissues may serve as molecular archives. Indeed, EPs are released by cells prior to tissue fixation and thus encapsulate biomolecular cargo that reflects the pre-fixation physiological state of the tissue. Once fixation occurs, EPs, particularly membrane-enclosed EVs and protein-dense NVEPs may become immobilized within the extracellular matrix or interstitial spaces. The physical structure (membrane, aggregation) of EPs confers partial protection of their contents from enzymatic degradation. Accumulating evidence suggests that EP-associated nucleic acids may be better preserved than bulk intracellular RNA during fixation and long-term storage [46,47].

The possibility that EPs isolated from FFT retain molecular information that is otherwise inaccessible in fixed tissues represents a conceptual shift in archival tissue analysis. Rather than viewing formalin fixation solely as a barrier to molecular studies, EPs may serve as preserved molecular “time capsules,” protecting short RNAs and other cargo from the most deleterious effects of fixation chemistry. Previous studies have primarily focused on isolating RNA or protein directly from FFPE tissue lysates. Far fewer investigations have addressed whether intact EPs can be recovered from fixed tissues and whether their RNA cargo remains informative. Addressing this gap is critical for unlocking the full translational potential of archival pathology specimens [48].

In this study, we sought to determine whether EPs could be isolated from formalin-fixed tissues and whether their RNA cargo retains biologically meaningful signatures. To address this, we analyzed five distinct types of formalin-fixed human tissues—placenta, heart, ovary, stomach, and gall bladder—representing diverse cellular compositions and metabolic states. The EPs were isolated from leftover discarded formalin-fixed tissues without reliance on freezing, demonstrating a practical workflow applicable to formalin fixed archival specimens. Small RNA recovered from these EPs was subjected to high-throughput sequencing using the Illumina small RNA library preparation protocol and sequenced on the Illumina NextSeq 2000 platform. Sequencing reads were mapped to miRBase [49] to identify human small RNAs and to Kraken-based databases for taxonomic classification of microbial sequences, enabling simultaneous assessment of host- and microbe-derived RNA content.

By integrating EP isolation with small RNA sequencing, this study aims to (i) establish the feasibility of recovering EP-associated RNA from fixed tissues, (ii) characterize the composition of small RNAs preserved within FFT-derived EPs, and (iii) lay the groundwork for using archival pathology specimens in EP-based molecular and translational research.

## 2. Materials and Methods

### 2.1. Ethics Statement

This study was conducted in accordance with Institutional Review Board (IRB) principles and approved by the IRB Committees of New York Medical College (NYMC) and Westchester Medical Center (WMC), Valhalla, NY, USA. After completion of clinical procedures, leftover tissues designated for disposal were deidentified and collected prior to discarding, in accordance with the approved IRB protocol.

### 2.2. Specimen Collection

Surgical specimens including placenta (*n* = 1), heart (*n* = 1), ovary (*n* = 1), stomach (*n* = 2), and gallbladder (*n* = 3) marked for “discard” were collected. Prior to collection, tissues were immersed in 10% neutral buffered formalin and stored at room temperature for approximately three weeks at WMC. Upon transfer to NYMC, tissues remained in formalin and were kept at room temperature until use, corresponding to 83–196 days from initial collection at WMC to processing at NYMC.

### 2.3. RNA Isolation and Sequencing

Total RNA was extracted using TRIzol (1 mL per 50–100 mg tissue) following standard protocol [50]. Small RNA libraries were prepared using the NEXTFLEX Small RNA Sequencing Kit v4 (NOVA-5132-31, Revvity, Inc., Waltham, MA, USA) according to the manufacturer’s instructions, including 3′ adapter ligation, cleanup, 5′ adapter ligation, and cDNA synthesis. Although the kit is optimized for small RNA enrichment, the library preparation workflow was modified to allow recovery of longer RNA inserts (~150 bp), enabling broader characterization of RNA species present in the samples. Libraries were sequenced using paired-end chemistry on an Illumina NextSeq 2000 platform (Illumina, Inc., San Diego, CA, USA). Paired-end sequencing was performed for superior read alignment because aligners use long-range positional information for stitching together contigs, assembling complex genomes with repetitive elements, and improving accuracy. RNA from frozen rat brain was included as an external biological reference control to evaluate EP isolation performance and RNA recovery in a non-formalin-fixed mammalian tissue context. While this introduces a species difference, it served as a technical comparator rather than a direct biological comparison.

### 2.4. Bioinformatic Analysis Human and Microbial Gene Expression

Initial alignment of sequencing reads to the human genome showed no detectable mapping. Broader taxonomic screening using NCBI BLAST v2.16.0 against the NR database indicated that the reads were predominantly of microbial origin. To further characterize these sequences, we used Kraken v2.17.1 [51] with the UHGG v2.0.2 database under default parameters. Paired-end FASTQ v0.12.1 files were processed using Kraken2, and taxonomic assignments were summarized using the generated Kraken report files. Reads classified as “unclassified” represent sequences that could not be assigned to any taxonomic node within the reference database using the Kraken2 classification framework.

### 2.5. Data Availability

The data discussed in this manuscript have been deposited in NCBI’s Gene Expression Omnibus (GEO) and are accessible through the GEO Series accession number GSE325844 (https://www.ncbi.nlm.nih.gov/geo/query/acc.cgi?acc=GSE325844, accessed on 7 July 2026).

### 2.6. Transmission Electron Microscopy (TEM)

Extracellular vesicle morphology was examined using transmission electron microscopy (TEM). Samples were applied onto carbon-coated copper grids and allowed to adsorb briefly. Excess liquid was removed, and samples were negatively stained with 1% uranyl acetate to enhance contrast. After air-drying, grids were visualized using a JEOL transmission electron microscop (JEOL USA, Inc., Peabody, MA, USA). Representative images were acquired to assess vesicle morphology and structural integrity.

### 2.7. Nanoparticle Tracking Analysis (NTA)

Particle size distribution and concentration were analyzed using a ZetaView (ZetaView PMX110, Particle Metrix, Mebane, NC, USA). Samples were diluted in 0.1× particle-free PBS to an appropriate concentration (1:1000) before measurement. Analysis was performed according to the manufacturer’s instructions, and recordings were acquired in triplicates per sample to ensure reproducibility. Data were processed using ZetaView software v8.04.02 to determine particle size distribution and concentration.

## 3. Results

### 3.1. Isolation of Extracellular Particles (EPs) from Formalin Fixed Tissues (FFT)

EPs were isolated from placenta, heart, ovary, stomach, and gallbladder tissues using our previously described protocols [52]. Briefly, small pieces of FFT (478–521 mg; Table 1) were finely chopped and digested with collagenase III [52]. Samples were clarified by sequential centrifugation at 500× *g*, 2500× *g*, and 12,000× *g* to remove cells and debris prior to EP isolation.

**Figure 1:**
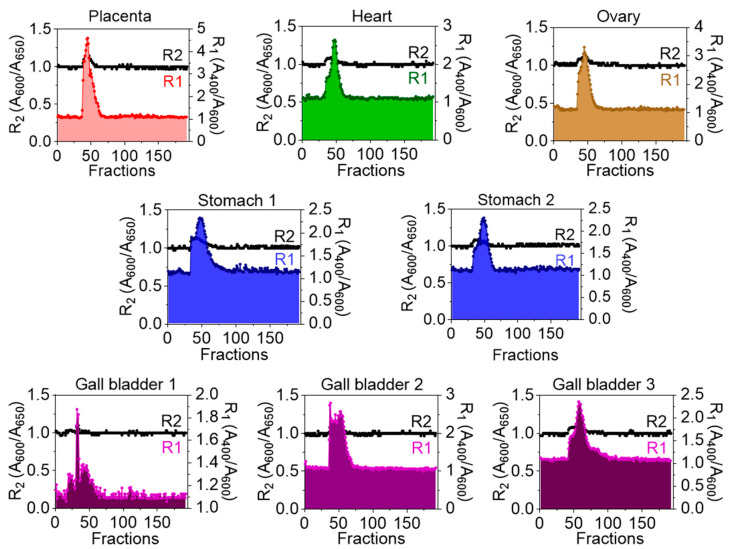
UV–Vis spectroscopy analysis of PPLC analytes from FFT-derived extracellular particles (EPs): Graph depicts the R1 and R2 absorbance ratios for each FFT-derived EP sample, where R1 = A400/A600 (right axis) and R2 = A600/A650 (left axis). These ratios reflect relative contributions of particle size, composition, and optical density within PPLC-resolved analytes.

The clarified supernatants were loaded onto a 50 × 0.5 cm Sephadex G-50 size-exclusion column and purified using the PPLC system as previously described [52,53]. Fifty fractions (200 µL each) were collected, and three-dimensional UV–Vis spectra (230–650 nm) were recorded to generate R1 and R2 ratios [53]. In the visible range, R1 = A400/A600 and R2 = A600/A650 served as qualitative turbidity indices used concurrently to identify fractions likely to contain membrane-associated particles (Figure 1). Based on these spectral signatures, membrane-containing fractions were pooled and stored in aliquots at −80 °C until further analysis.

Protein concentrations ranged from 0.45–6.66 µg/µL for membrane-containing fractions and 0.02–0.41 µg/µL for non-membrane fractions (Table 1). Given the spectral profiles (Figure 1) and the markedly lower protein content of non-membrane fractions, all subsequent analyses focused on the membrane-containing EP fractions.

### 3.2. Characterization of the Physical Properties of Extracellular Particles (EPs) from Formalin Fixed Tissues (FFT)

**Figure 2:**
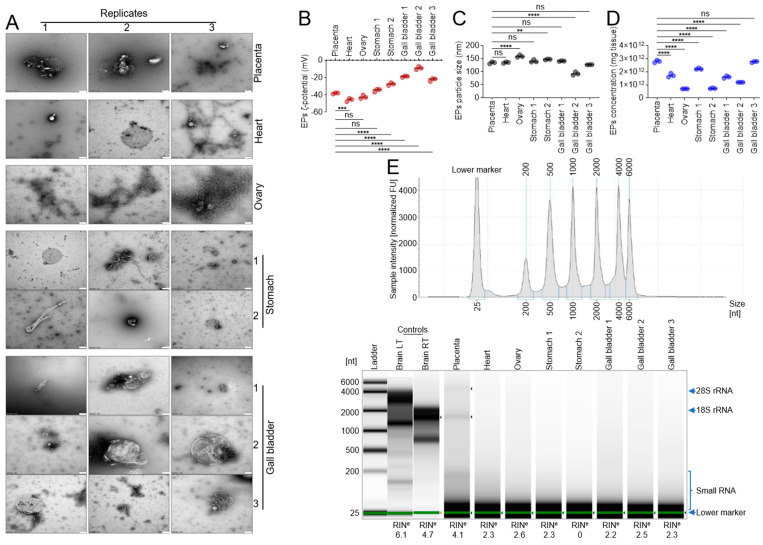
Physicochemical characterization of FFT-derived extracellular particles (EPs): (**A**) Representative transmission electron microscopy (TEM) image of FFT-derived EPs. (**B**) Zeta (ζ)-potential (mV) of FFT-derived EPs measured by nanoparticle tracking analysis (NTA; ZetaView). (**C**) Size distribution (nm) of FFT-derived EPs measured by NTA (ZetaView). (**D**) EP concentration normalized to tissue mass (mg tissue) measured by NTA (ZetaView). (**E**) Agilent RNA ScreenTape assay (Agilent 4200 TapeStation) showing the electronic ladder (top) and gel image with corresponding RINe values displayed below each lane (bottom). Scale bar (unclear text left bottom of each image) in panel (**A**) = 200 nm. Error bars represent the standard error of the mean (SEM) for panels (**B**–**D**). Statistical significance was determined by ordinary one-way ANOVA with Šídák’s multiple comparisons test. **** *p* < 0.0001, *** *p* < 0.0005, ** *p* < 0.01, ns = not significant.

Transmission electron microscopy revealed the presence of particles with heterogeneous sizes and morphologies (Figure 2A). Both intact and disrupted membrane-bound structures were observed, supporting our decision to focus subsequent analyses on membrane-containing fractions. Nanoparticle tracking analysis (NTA) was used to assess ζ-potential, particle size, and concentration (Figure 2B–D). The mean ζ-potential values for placenta, heart, ovary, stomach-1, stomach-2, gallbladder-1, gallbladder-2, and gallbladder-3 were −38.13 ± 0.70, −45.68 ± 1.93, −42.33 ± 2.12, −34.60 ± 1.84, −27.50 ± 1.66, −18.87 ± 0.93, −9.07 ± 1.88, and −22.44 ± 1.82 mV, respectively (Figure 2B). Mean particle sizes for the same tissues were 133.93 ± 3.96, 134.87 ± 2.54, 159.10 ± 4.97, 140.43 ± 5.45, 146.77 ± 1.76, 140.33 ± 1.40, 92.40 ± 6.74, and 126.43 ± 0.64 nm, respectively (Figure 2C). Mean particle concentrations were 2.8 × 10^12^ ± 1 × 10^11^, 1.7 × 10^12^ ± 1.5 × 10^11^, 7 × 10^11^ ± 1 × 10^10^, 2.23 × 10^12^ ± 5.8 × 10^10^, 7.37 × 10^11^ ± 3.79 × 10^10^, 1.6 × 10^12^ ± 1 × 10^11^, 1.2 × 10^12^ ± 0, and 2.77 × 10^12^ ± 5.77 × 10^10^ particles per mg tissue, respectively (Figure 2D).

RNA quality was assessed using the Agilent RNA ScreenTape assay on the Agilent 4200 TapeStation (Agilent Technologies, Inc., Santa Clara, CA, USA) system. The gel image displays the separation profile for each sample, including 28S and 18S rRNAs, small rRNAs, and the lower marker, with RNA Integrity Number equivalent (RIN^e^) values shown below each lane (Figure 2E). The RNA profiles of FFT-derived EPs were distinct from those of RNA isolated from frozen rat brain tissue. As expected, FFT-EP RNA consisted primarily of short RNA fragments 25–50 nt, whereas rat brain RNA ranged from ~150 to 4000 nt (Figure 2E). It remains difficult to determine whether RNA extracted from all FFT specimens was degraded, as EPs naturally contain small RNAs that are protected from extracellular RNases.

### 3.3. Sequencing Data Output

Small RNA libraries were generated from FFT-derived EPs using the Illumina small RNA protocol and sequenced on the Illumina NextSeq 2000 platform. Reads were first mapped to miRbase [54]. Sequences that did not align with any known region of the human genome were classified as unclassified, comprising 63–95% of total RNA reads. To assess microbial contributions, reads were also analyzed using Kraken, which identified microbial sequences and RNAs aligning to known microbial taxa. Overall, 81% of reads were unclassified, while 18.5% mapped to bacterial sequences (Table 2, Table S1). The overall value of 18.5% bacterial mapping represents the proportion of reads assigned to bacterial taxa when calculated across the entire dataset based on total read counts pooled from all samples.

Sequencing of the eight FFT-derived EP samples yielded reads mapping to 6127 taxonomic entries. Among all tissues, the gallbladder libraries exhibited the lowest proportion of unclassified sequences, with 11.73%, 39.57%, and 37.04% of nucleotide reads assigned to microbial taxa. In contrast, the stomach-2 library showed the highest percentage of unclassified sequences (95.95%) (Table 3). Artifacts refer specifically to adapter dimers and very short inserts that were removed during preprocessing. Non-artifact, which are reads that passed adapter trimming and length filtering criteria were analyzed. The observation of 100% non-artifact reads in gallbladder-2 and gallbladder-3 reflects that, after preprocessing, all retained reads in these samples passed filtering thresholds and no adapter-dimer or ultra-short contaminant sequences were detected. This does not imply a biological absence of variation, but rather complete removal of technical artifacts during preprocessing for those specific libraries.

The highest number of microbial species identified in FFT-derived EPs was observed in the following order: gallbladder-1 > ovary > gallbladder-2 > heart (Table 4). These findings highlight substantial variability in microbial representation across tissues and underscore the influence of fixation, storage conditions, and tissue-specific factors on the detectability and classification of microbial sequences in FFT-derived EPs.

**Figure 3:**
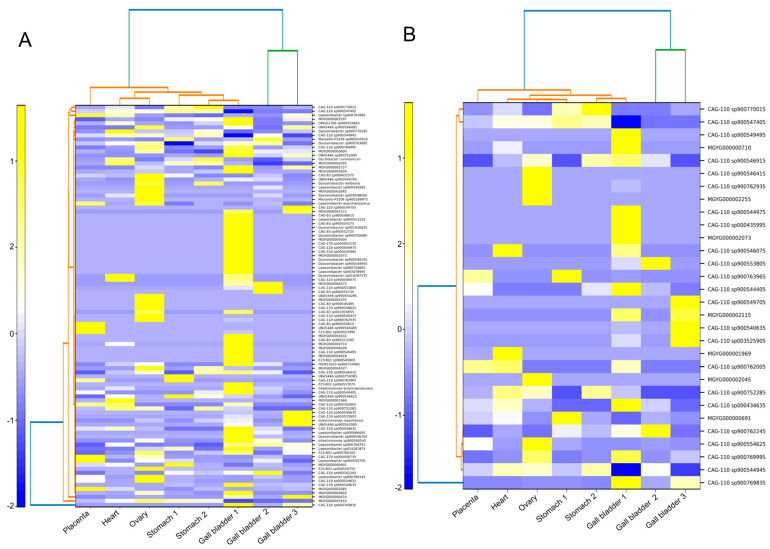
Relative abundance of microbial taxa detected in FFTderived EPs. (**A**) Heatmap showing the relative abundance of the top 100 most abundant microbial taxa across all FFT-derived EP samples. (**B**) Heatmap showing the relative abundance of the top 30 most abundant microbial taxa across the same samples.

**Figure 4:**
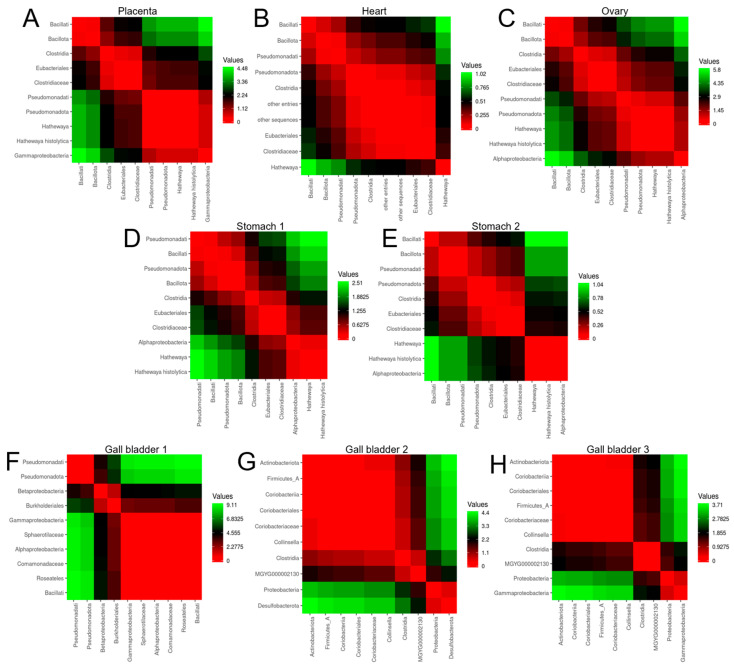
Relative abundance of the top 10 microbial taxa within each FFT-derived EP sample. (**A**–**H**) Heatmap showing the relative abundance of the top 10 most abundant microbial taxa identified within each FFT-derived EP sample. (**A**) Placenta, (**B**) Heart, (**C**) Ovary, (**D**,**E**) Stomach, (**F**–**H**) Gall bladder. Each column represents an individual FFT-derived EP preparation, and each row represents one of the top 10 taxa enriched in that sample.

We identified the top 100 and top 30 microbial sequences associated with FFT-derived EPs (Figure 3A,B). Among the top 30 taxa, *Firmicutes bacterium* and *Clostridiaceae bacterium* were consistently represented (Figure 3B). We also examined the top 10 microbial sequences for each sample (Figure 4A–H). *Bacillati* and *Pseudomonadota* were present in all samples except gallbladder-2 and gallbladder-3 (Figure 4G,H). Similarly, *Clostridia* and *Clostridiaceae* were detected in all samples except the gallbladder tissues, whereas *Comamonadaceae* sequences were uniquely enriched in the gallbladder samples (Figure 4F–H). These patterns highlight both shared microbial signatures across FFT-derived EPs and tissue-specific microbial enrichment.

**Figure 5:**
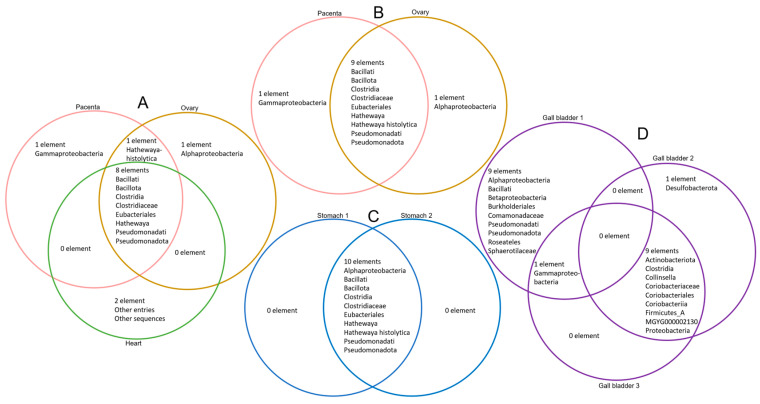
Shared and unique microbial taxa among FFT-derived EP samples. Venn diagram illustrating the overlap of the top 10 most abundant microbial taxa across FFT-derived EP samples, highlighting microbial elements that are common or unique to each sample. (**A**) Placenta versus Ovary versus Heart, (**B**) Placenta versus Ovary, (**C**) Stomach 1 versus Stomach 2, (**D**) Gall bladder 1 versus Gall bladder 2 versus gall bladder 3.

Venn analyses identified shared and unique microbial elements across FFT-derived EP datasets (Figure 5A–D). Eight taxa—*Bacillati*, *Bacillota*, *Clostridia*, *Clostridiaceae*, *Eubacteriales*, *Hathewaya*, *Pseudomonadati*, and *Pseudomonadota*—were common to the placenta, ovary, and heart (Figure 5A). Nine taxa—*Bacillati*, *Bacillota*, *Clostridia*, *Clostridiaceae*, *Eubacteriales*, *Hathewaya*, *Hathewaya histolytica*, *Pseudomonadati*, and *Pseudomonadota*—were shared between the two reproductive tissues, the placenta and ovary (Figure 5B). In the stomach samples, all 10 taxa—*Alphaproteobacteria*, *Bacillati*, *Bacillota*, *Clostridia*, *Clostridiaceae*, *Eubacteriales*, *Hathewaya*, *Hathewaya histolytica*, *Pseudomonadati*, and *Pseudomonadota*—were common to both stomach tissues (Figure 5C). In the gallbladder datasets, one taxon (*Gammaproteobacteria*) was shared between gallbladder-1 and gallbladder-3, whereas nine taxa (*Actinobacteriota*, *Clostridia*, *Collinsella*, *Coriobacteriaceae*, *Coriobacteriales*, *Coriobacteriia*, *Firmicutes A*, *MGYG000002130*, and *Proteobacteria*) were shared between gallbladder-2 and gallbladder-3. Notably, no taxa were common between gallbladder-1 and gallbladder-2 (Figure 5D).

## 4. Discussion

The primary goal of this study was to determine whether extracellular particles (EPs) can be isolated from leftover discarded formalin-fixed tissues (FFT) and to characterize the cargo composition of FFT-derived EPs to assess their potential utility for clinical or research applications. At the time of EP isolation, tissues had been stored in 10% neutral buffered formalin at room temperature for 83–196 days. We demonstrate for the first time that EPs are present in leftover discarded FFT and that RNA can be recovered from these EPs. However, RNA-Seq analysis revealed that formalin fixation and prolonged room-temperature storage resulted in RNA profiles dominated by unclassified and microbial sequences. Reads that did not map to any region of the human genome were categorized as unclassified and accounted for approximately 81% unclassified total RNA reads and 18.5% of bacterial RNA reads across tissues.

Sequencing analysis of FFT-derived EPs provides an opportunity to interrogate archived specimens that are otherwise inaccessible for molecular studies. Although our samples originated from different tissue types, the overall profiles of enriched RNAs and microbial sequences were broadly similar. While little is known about microbial communities that may inhabit leftover discarded FFT, our findings offer rare insight into microbial signatures associated with such specimens. We identified microbial sequences mapping to both known and unknown taxa, with unclassified reads comprising 91% (Placenta), 95% (Heart), 87% (Ovary), 92% (Stomach-1), 96% (Stomach-2), 88% (Gallbladder-1), 60% (Gallbladder-2), and 63% (Gallbladder-3). In contrast, bacterial sequences represented 9%, 4%, 12%, 8%, 4%, 12%, 39%, and 36% of reads in the same tissues, respectively.

It is not unexpected that most sequences recovered from FFT-derived EPs were unclassified, given that the Earth is estimated to contain 10^11^–10^12^ microbial species, of which 99.999% remain undiscovered [55]. Whether the sequences identified here represent true microbial elements or fixation-induced artifacts remains unclear, especially given the absence of process and reagent controls. Formalin is known to introduce sequence artifacts in next-generation sequencing (NGS) datasets, and some unclassified reads may reflect such effects.

Although formaldehyde-mediated cross-linking begins immediately upon exposure, the timing and extent of its impact on RNA integrity remain poorly understood. Our study shows that leftover discarded FFT can provide large volumes of biospecimens for EP isolation; however, it remains uncertain whether host genomic information can be reliably obtained from FFT-derived EPs or whether microbial sequences detected in these samples reflect biological reality or fixation-related artifacts.

## 5. Conclusions

Our results suggest that leftover discarded FFTs contain extracellular particles (EPs) from which RNA can be recovered and subjected to RNA-Seq analysis. However, the majority of RNAs isolated from available FFT-derived EPs were unclassified and therefore did not yield comprehensive or interpretable transcriptomic information. This limitation underscores the need for access to properly collected, flash-frozen, or otherwise well-preserved specimens to ensure RNA integrity and analytical rigor. These findings highlight the importance of establishing institutional or multi-departmental biobanking infrastructure capable of prospectively gathering, annotating, and storing sufficient high-quality samples to support robust EP and exRNA studies. Of note, this study has some limitations. One fixation method from a single institution was examined and the conditions of specimen collection/processing were not evaluated; there was an absence of reagent control and small sample sizes. These limitations should be considered while interpreting the findings of this study.

## Figures and Tables

**Table 1:** Characteristics of the tissues used for isolation of EPs.

Surgical Specimen	Tissue Description	Number of Days in Formalin	Weight of Tissue Used for Isolation of EPs (mg)	EVs Protein Concentration (µg/µL)	NVEPs Protein Concentration (µg/µL)
Placenta	A portion of placenta	166	508	6.66	0.07
Heart	A mass near the left ventricular apex	196	478	2.38	0.06
Ovary	A large solid nodule	194	514	3.31	0.41
Stomach-1	A portion of stomach	167	495	1.79	0.05
Stomach-2	A portion of stomach	167	490	1.25	0.09
Gall bladder-1	Half of Polypoid nodule	83	521	0.45	0.02
Gall bladder-2	A portion of gall bladder	83	512	3.85	0.26
Gall bladder-3	A portion of gall bladder	83	478	1.69	0.12

**Table 2:** Small RNASeq data showing microbial enrichment in EPs isolated from FFT.

Placenta EPs		Heart EPs		Ovary EPs		Stomach-1 EPs		Stomach-2 EPs		Gall Bladder-1 EPs		Gall Bladder-2 EPs		Gall Bladder-3 EPs	
% of Reads Covered by the Clade Rooted at This Taxon	Scientific Name	% of Reads Covered by the Clade Rooted at This Taxon	Scientific Name	% of Reads Covered by the Clade Rooted at This Taxon	Scientific Name	% of Reads Covered by the Clade Rooted at This Taxon	Scientific Name	% of Reads Covered by the Clade Rooted at This Taxon	Scientific Name	% of Reads Covered by the Clade Rooted at This Taxon	Scientific Name	% of Reads Covered by the Clade Rooted at This Taxon	Scientific Name	% of Reads Covered by the Clade Rooted at This Taxon	Scientific Name
90.51	Unclassified	95.21	Unclassified	87.39	Unclassified	91.58	Unclassified	95.95	Unclassified	88.27	Unclassified	60.43	Unclassified	62.96	Unclassified
9.49	Root	4.79	Root	12.61	Root	8.42	Root	4.05	Root	11.73	Root	39.57	Root	37.04	Root
9.04	Cellular organisms	3.71	Cellular organisms	12.37	Cellular organisms	8.1	Cellular organisms	3.68	Cellular organisms	11.69	Cellular organisms	38.7	Bacteria	35.84	Bacteria
8.97	Bacteria	3.65	Bacteria	12.28	Bacteria	7.97	Bacteria	3.61	Bacteria	11.58	Bacteria	7.79	*Actinobacteriota*	7.05	*Actinobacteriota*
5.15	*Bacillati*	1.59	*Bacillati*	6.68	*Bacillati*	3.56	*Pseudomonadati*	1.58	*Bacillati*	9.57	*Pseudomonadati*	7.57	*Firmicutes_A*	6.93	*Coriobacteriia*
4.79	*Bacillota*	1.44	*Bacillota*	6.19	*Bacillota*	3.32	*Bacillati*	1.39	*Bacillota*	9.09	*Pseudomonadota*	7.51	*Coriobacteriia*	6.93	*Coriobacteriales*
3.54	*Clostridia*	1.35	*Pseudomonadati*	4.84	*Clostridia*	3.08	*Pseudomonadota*	1.39	*Pseudomonadati*	5.51	*Betaproteobacteria*	7.51	*Coriobacteriales*	6.82	*Firmicutes_A*
3.06	*Eubacteriales*	1.18	*Pseudomonadota*	4.13	*Eubacteriales*	2.94	*Bacillota*	1.21	*Pseudomonadota*	3.75	*Burkholderiales*	7.31	*Coriobacteriaceae*	6.69	*Coriobacteriaceae*
2.99	*Clostridiaceae*	1.12	*Clostridia*	4.01	*Clostridiaceae*	2.41	*Clostridia*	1.13	*Clostridia*	0.96	*Gammaproteobacteria*	7.31	*Collinsella*	6.69	*Collinsella*
1.6	*Pseudomonadati*	1.06	Other entries	2.9	*Pseudomonadati*	2	*Eubacteriales*	1.04	*Eubacteriales*	0.8	*Sphaerotilaceae*	6.56	*Clostridia*	5.39	*Clostridia*

**Table 3:** Microbial Reads associated with FFT-derived EPs.

Specimen	Mean High Quality Read Pairs	Non-Artifacts ^a^	Unclassified	Microbial Pairs
Placenta	12,615,237	3,108,216 (24.63%)	2,813,211 (90.5%)	295,005 (9.49%)
Heart	34,440,465	8,019,895 (23.29%)	7,636,071 (95.21%)	383,824 (4.79%)
Ovary	18,605,859	6,274,541 (33.73%)	5,483,537 (87.39%)	791,004 (12.61%)
Stomach-1	6,855,946	1,805,981 (26.34%)	1,653,863 (91.58%)	152,118 (8.42%)
Stomach-2	13,818,468	3,267,615 (23.64%)	3,135,430 (95.95%)	132,185 (4.05%)
Gall bladder-1	104,544,227	37,373,817 (35.75%)	32,988,688 (88.27%)	4,385,129 (11.73%)
Gall bladder-2	21,611,324	21,611,324 (100%)	13,060,112 (60.43%)	8,551,212 (39.57%)
Gall bladder-3	4,366,940	4,366,940 (100%)	2,749,485 (62.96%)	1,617,455 (37.04%)

^a^ Artifacts refer to Adapter dimers/very short inserts. The taxonomy has 6127 entries. There are 697,010 microbes in the database.

**Table 4:** Microbial diversity associated with FFT-derived EPs.

Level	Placenta	Heart	Ovary	Stomach-1	Stomach-2	Gall Bladder-1	Gall Bladder-2	Gall Bladder-3
Species	878	997	1087	769	772	1386	1031	691
Genus	729	794	843	660	661	983	821	588
Family	193	197	203	183	186	211	203	180
Order	87	86	89	83	85	89	88	84
Class	33	33	33	31	33	33	33	32
Phylum	24	24	24	23	24	24	24	23

## Data Availability

The original contributions presented in this study are included in the article/Supplementary Material. Further inquiries can be directed to the corresponding authors.

## Supplementary Materials

The following supporting information can be downloaded at: https://www.mdpi.com/article/10.3390/biom16070993/s1, Table S1: SupplementalDataSet_All samples small RNASeq Data.
